# Computation of Traffic Time Series for Large Populations of IoT Devices

**DOI:** 10.3390/s19010078

**Published:** 2018-12-26

**Authors:** Mikel Izal, Daniel Morató, Eduardo Magaña, Santiago García-Jiménez

**Affiliations:** 1 Electrical, Electronic and Communications Engineering Department, Universidad Pública de Navarra, 31006 Pamplona, Spain; daniel.morato@unavarra.es (D.M.); eduardo.magana@unavarra.es (E.M.); santiago.garcia@unavarra.es (S.G.-J.); 2 Smart Cities Institute, Universidad Pública de Navarra, 31006 Pamplona, Spain

**Keywords:** IoT, network traffic, monitoring, DDoS, packet classification

## Abstract

The Internet of Things (IoT) contains sets of hundreds of thousands of network-enabled devices communicating with central controlling nodes or information collectors. The correct behaviour of these devices can be monitored by inspecting the traffic that they create. This passive monitoring methodology allows the detection of device failures or security breaches. However, the creation of hundreds of thousands of traffic time series in real time is not achievable without highly optimised algorithms. We herein compare three algorithms for time-series extraction from traffic captured in real time. We demonstrate how a single-core central processing unit (CPU) can extract more than three bidirectional traffic time series for each one of more than 20,000 IoT devices in real time using the algorithm DStries with recursive search. This proposal also enables the fast reconfiguration of the analysis computer when new IoT devices are added to the network.

## 1. Introduction

The Internet Protocol (IP) provides connectivity to millions of smart and autonomous devices. They range from small health monitors, ambient sensors, and location notification devices to seismic sensors, traffic cameras, and generic computers. The smart devices represent a new wave in network-connected elements that is expected to surpass the number of computer hosts. Different predictions estimate between 20 to 200 billion of these devices by 2020 [1,2].

The service architecture for each type of device differs, but a centralised information-collecting element is typical. Deployed devices typically communicate with a central collector using virtual private networks offered by mobile communication companies or local Internet service providers (ISPs) [1]. An example scenario would be energy consumption-metering devices deployed in households; they communicate with the central office using power lines. Other examples include sensors deployed in remote wind power production towers that communicate using cellular networks, temperature and pressure sensors for weather forecasting, and location devices for fleet tracking logistics [3]. The Internet of Things (IoT) ecosystem offers a plethora of examples of large populations of small sensing devices that collect information and send them to centralised hosts.

**Figure 1:**
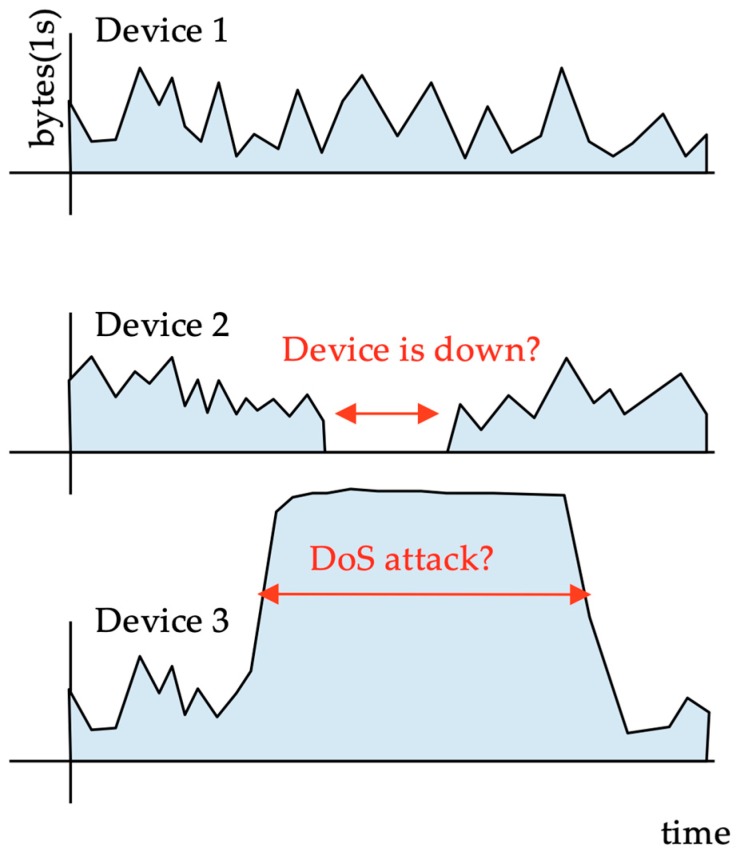
A disconnected device is detected using the traffic it generates.

The process of monitoring the availability of these ‘things’ is a difficult task, owing to the large number of devices. It can be achieved by active or passive monitoring. Any type of check that actively communicates with the devices would inject a large amount of traffic into the access networks, whereas passive monitoring techniques do not add any load to the network or the devices. Passive monitoring can be based on a time-series analysis of network traffic from each device. These traffic time series can be used to verify device liveliness by detecting periods of network traffic silence (Figure 1). For cellular operators, traffic time series are fundamental for evaluating traffic patterns from different types of IoT devices. It is noteworthy that these devices typically employ a cellular network [4], competing for resources with smartphone users [5]; therefore, network dimensioning requires traffic profiles for different cellular user types. 

Traffic time series can also be used for security monitoring. The analysis of anomalies in device traffic patterns can be used to detect erratic behaviour from the smart devices due to malfunctions or security violations [6]. Currently, the IoT ecosystem offers many devices with low security, and has already been used for the distributed denial-of-service (DDoS) attacks [7]. Monitoring the network activity from these devices to the centralised hosts (or to any other destination) is critically important for providing early intrusion detection.

Creating a per-device time series requires classifying each network packet when hundreds of thousands of classes are defined. Each packet could belong to only one class; therefore, it is considered in the time series for one device, or multiple time series could be created for each device. For example, all of the traffic that a device sends to its configured centralised collector could be recorded in a time series while separately accounting for all the traffic it sends to other destinations in a different time series (to detect anomalies). Therefore, time-series extraction requires the multi-label classification of packets, where a given packet may be assigned to several classes: one for each time series for which it has to be accounted.

Most sensors collect little and infrequent information; therefore, the network traffic they create presents a low bitrate. Low-rate information includes, for example, the location information sent from sensors installed in cars from a rental company. However, other sensors produce higher bitrates, for example, seismic sensors or surveillance cameras. In a traffic aggregation point or a location close to a collector in a large population of devices, millions of packets per second are expected [8], implying several gigabits of traffic per second. 

Creating hundreds of thousands of time series based on source and destination when each packet can account for more than one class is not a trivial problem. This level of monitoring is typically performed by collecting NetFlow statistics from IP routers and postprocessing those flows to create the time series [9,10,11]. However, NetFlow monitoring presents time-resolution limitations. When a flow has finished, aggregated counters are provided. While the flow is active, the periodic dumps of all of the flows in memory of the networking device are sent to the NetFlow collector. Owing to the large amount of active flows, this collection has a periodicity of several minutes, thus losing all of the details below this scale. Most analysis and prediction algorithms require several measurement points in the time series, which would require several minutes to be collected from flow records. Reaction times in the order of several minutes are not appropriate for critical devices such as health monitoring sensors or probes in a nuclear plant. However, reducing the period of active flow collection in NetFlow statistics implies a higher load on the networking equipment, making it unfeasible.

The objective of this work is to validate that time-series extraction directly from a network packet stream can be performed sufficiently fast, and for a sufficiently large class set, to cope with the expected IoT scenarios. The traffic stream can be obtained by mirroring the packet flow at a network switch. This is a common functionality offered by most enterprise class switches. We focus on the extraction of traffic volume time series, namely the byte or packet counters, from a passive monitoring probe. Our test probe processes several gigabits per second of traffic in real time, providing the time series with a resolution better than one second.

Packet classification algorithms are a central part of this system. They assign each packet to a single class or multiple classes of equivalence. Packet classification is a well-studied problem [12,13,14]. If the number of classes is not large, a simple linear search for every packet over the class list is typically sufficient. However, depending on the traffic arrival rate and the computing power, above a certain number of classes, reviewing every class in sequence could consume too much time. Then, the analysis machine cannot cope with traffic at line rate without splitting the work among several central processing unit (CPU) cores, thereby increasing hardware costs. Several algorithms have been proposed and used to reduce the number of classes to visit [13,14]. Within hardware systems, optimal performance can be achieved using structures based on ternary content addressable memories (TCAMs) [15]. They can perform comparisons in parallel with all of their content cells. However, they incur high development costs for the ad-hoc hardware solution. 

Software-based packet classification solutions sort the classes into binary search trees (also called “tries”). As classes typically involve IP sources and destination addresses, a hierarchy of search tries is used: first for the destination address, and subsequently for the source address. This hierarchy enables identifying the location of the relevant classes in only a few operations. The complexity in classification grows with the number of bits in the input classification information. However, this is at the expense of algorithm complexity and the memory that is required to store the search tries’ structure. Probabilistic classification structures such as neural networks or Bloom filters, which are quite common in other classification scenarios, are not appropriate for the problem at hand. They have been used when high uncertainty in the classification is present or when the number of possible classes is very small [16,17]. On the contrary, the classification for time-series extraction in an IoT scenario is deterministic: it is based entirely on source and destination addresses, and it must classify packets when a large number of classes is defined. Neural networks and Bloom filters, being probabilistic, can produce wrong classification results. Failure in classification in one of these structures for a pair of source and destination network addresses does not happen for random independent packets, but rather for every packet in a time series between the same pair of addresses; therefore null or flat-line time series would result. On the other hand, both techniques require at least as many computing elements as the number of possible classes. For neural networks, at least one neuron is required for each possible class. Using Bloom filters, at least one filter is required for each class. Both structures can be implemented in parallel hardware architectures; however, in software implementations, they present a linear complexity with the number of classes, which in the present scenario we will show can reach hundreds of thousands of classes.

A survey of packet classification algorithms can be found in [12]. They are proposed for quality of service and security filtering scenarios. We herein evaluate how these algorithms apply to the time-series computation problem when the number of classes is as large as that expected in IoT scenarios. 

To calculate the traffic time series for monitoring purposes, a packet has to be assigned to every class to which it belongs. The algorithm cannot stop whenever it obtains the best matching class, but it has to search over all the classes that may apply. We have selected three algorithms that can be used for multi-label classification: the linear list of rules search, the simple hierarchical tries [12], and the set-pruning tries [12]. Other popular algorithms such as the grid-of-tries [18] have to be discarded, because they do not comply with the multiclass requirement. 

This work demonstrates that the hierarchical tries with recursive search can be used to build passive monitoring systems that can monitor tens of thousands of IoT devices in real time, with fast reconfiguration when devices are added or removed.

In Section 2 of this paper, the network scenario is presented, selected algorithms are described, and the methodology for performance evaluation is explained. In Section 3, the algorithms are compared using several performance metrics. Section 4 concludes the paper.

## 2. Methodology

### 2.1. Scenario

We consider an ISP offering network connectivity to IoT devices. These devices are separated into different groups. The network provider assigns addresses to the devices in order to create these groups. For example, a group could contain all of the devices in one building (or in the same region), or all of the devices with the same type of sensor. For some IoT applications (e.g., ubiquitous sensors), these groups may contain hundreds of thousands of devices, each one with an individual network address.

Network addresses are a sequence of bits that is used as a network locator, and are assigned according to the network protocol (IPv4, IPv6…). Different protocols use different numbers of bits in the sequence (32 in IPv4, 128 in IPv6...) or even variable length addresses. A given host, such as the controller for a group of devices, will be assigned a well-known network address. Groups of devices in the same network will be assigned an address in the same so-called subnetwork, namely a set of addresses with the same prefix bits. The first p bits in an address are called a prefix of size p.

**Figure 2:**
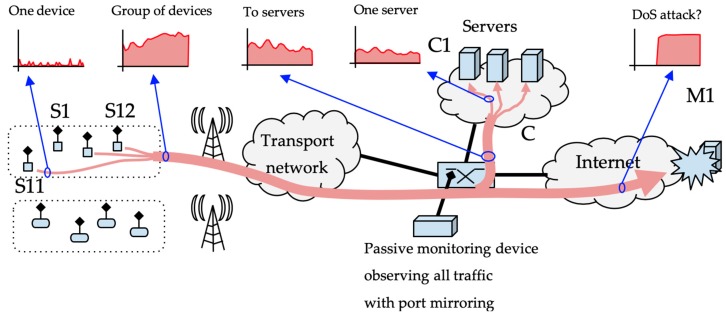
Network scenario with devices grouped by network address.

Monitoring traffic from a large population of IoT devices requires classifying traffic coming from or going to different single network addresses or network address prefixes. Figure 2 shows the scenario under consideration. Several groups of devices communicate with different destinations through the ISP’s network. This communication is bidirectional; the controllers may send requests to the devices, and the devices may send data to the controlling hubs. Monitoring this communication implies separating different views of the traffic. We can examine traffic from a single device or from a group of devices. We can be interested in traffic from a group of devices to one controlling server, or in the opposite direction from one controlling hub to a group of devices. It may be interesting to separate traffic from groups of devices to any address to detect whether devices are being used to perform denial-of-service attacks to Internet targets, or detect communications with suspected malware hubs.

An ISP monitoring system has to cope with this diversity of traffic flows. Every flow of interest can be described by a source and a destination. Every source and destination may be a single network address or a group of addresses given by a network address prefix with a length of p bits. We define a class as any combination of source and destination addresses in which the monitoring system is interested. A passive monitoring device that examines every network packet going through the ISP’s network has to decide whether the packet belongs to any possible class of interest.

We denote $C=\left\{C_{i}\right\}$, where $C_{i}=\left(s_{i},d_{i}\right)$ is the set of classes of interest for the monitoring system. The elements of C are our traffic-flow classes.

$s_{i}$ is a source prefix, and $d_{i}$ is a destination prefix. They may be prefixes, full addresses (a prefix of n bits), or the “any host” address (represented by a prefix with 0 bits).

S is the set of source prefixes of interest $S=\left\{s_{i}\right\}$. The number of elements in S is $N_{S}$. Similarly, D is the set of destination prefixes of interest $D=\left\{d_{i}\right\}$. The number of elements in D is $N_{D}$. C is the set of classes of interest. C is a subset of the Cartesian product SxD. 

The number of elements of C that can be used may be as large as $N_{S}\;x\;N_{D}$, in case we are interested in every possible source–destination combination. In a real scenario, only a subset of these combinations will have to be monitored, and the number of classes in the set will be $N_{C}<N_{S}\;N_{D}$.

As the number of addresses and prefixes of interest increases, it is more difficult for traffic-processing software to classify packets on a larger set of classes in real time.

### 2.2. Algorithms and Data Structures

We herein evaluate three popular algorithms for multi-label packet classification. The algorithms store the list of classes in different data structures. Every network packet can be matched against any class stored in these data structures. We compare the performance of these algorithms in scenarios with a large number of classes, $N_{C}$. We will measure the performance based on the packet processing speed and its memory footprint.

Table 1 contains the symbols describing the classes used in the data structure examples that follow. A packet may belong to several classes, i.e., a packet from S12 to C1 should be in classes one, three, and six.

#### A. Linear Search (LS) over the List of Classes

**Figure 3:**
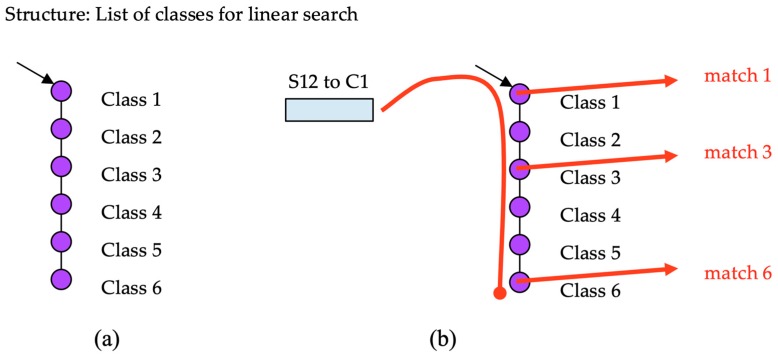
(**a**) Data structures and (**b**) search procedure in linear search (LS).

As a simple reference algorithm, using linear search (LS), every packet is evaluated sequentially against all of the $N_{C}$ classes in C as shown in Figure 3. The list of classes is stored in a simple linked list or in an array. The expected per-packet processing time depends on the number of classes $N_{C}$ as $O\left(N_{C}\right)$.

#### B. DStries with Recursive Search

A data structure called a DStrie [12] is built to store all of the classes. This structure is composed of several substructures called tries, which are created as follows.

**Figure 4:**
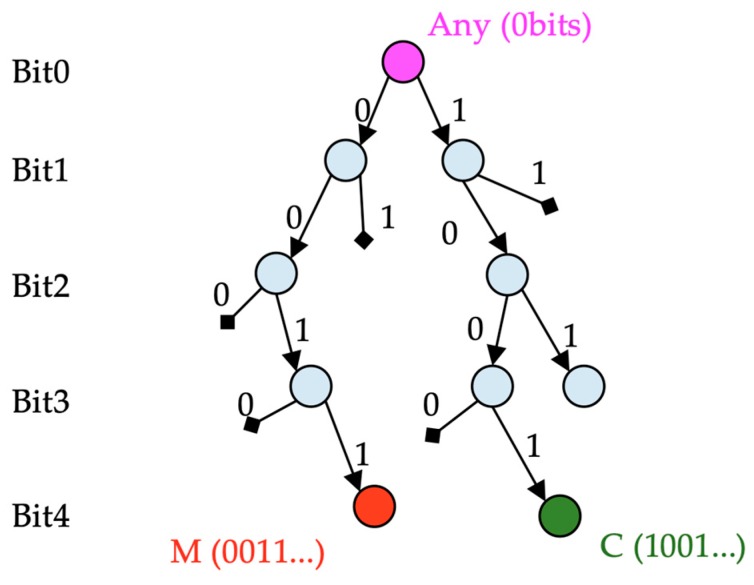
Destination prefix binary search tree (*trie*).

A binary search tree (trie) is a data structure that can store a set of network address prefixes. Prefixes are stored in a decision tree starting with the first bit of the address. For example, to store the binary prefix 0011, four nodes are created on the tree. The first bit is 0, the second is 0, the third is 1, and the fourth is 1. Data associated with prefix 0011 would be stored at the red node in Figure 4. Those associated with prefix 1001 would be stored at the green node in Figure 4, and those associated with the prefix of length 0 bits (representing any address) would be stored at the root node. Decision nodes that are not required to reach any of the stored prefixes are not in the tree. Every node in the tree is associated with a prefix, but that prefix may not be stored in the tree. For example, in Figure 4, the node for prefix 001 does not contain any stored information, but it exists because it is required to reach prefix 0011. Meanwhile, the node for prefix 010 is not in the tree. The tree is built by adding to all of the decision nodes that are required to reach the stored prefixes of the tree.

The DStrie data structure has to store classes with a source prefix and a destination prefix. It is composed of two levels of tries. The high-level trie stores destination prefixes. Every time a packet is examined, its destination address is searched for in the destination trie to find all of the destination prefixes that may apply to that address. A class also contains a source prefix; therefore, every node in the destination trie that stores a prefix also stores a pointer to a second-level trie containing the source prefixes. 

**Figure 5:**
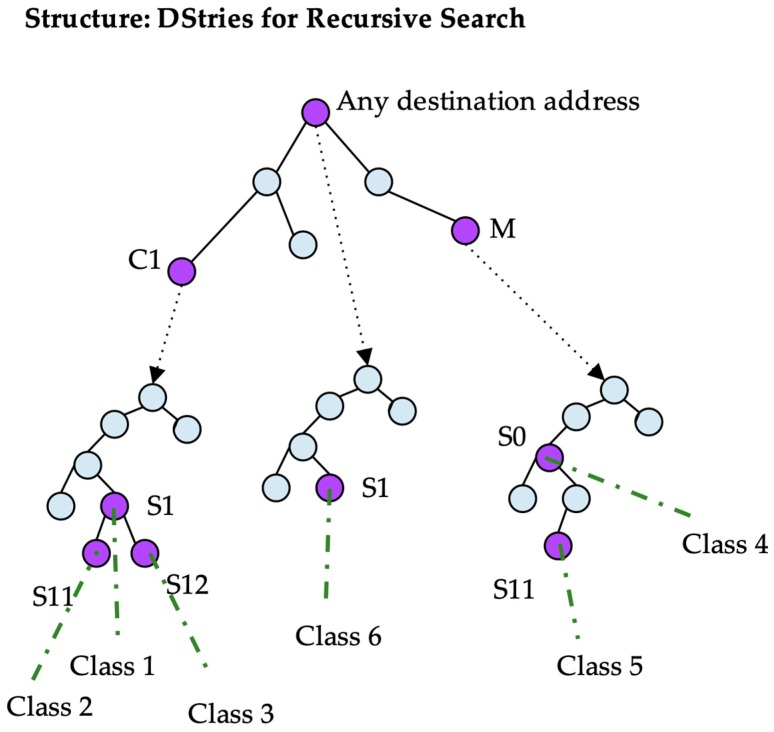
DStrie for recursive search example.

A given traffic class is stored in the node that is indicated by its source prefix in the source trie that is pointed to by the node in the destination trie corresponding to its destination prefix. An example is shown in Figure 5.

To classify a packet with a given source and destination address, first, the destination address is searched for in the destination trie. After the destination is found, the source is searched for in the corresponding source trie. A packet that verifies a destination node also verifies every destination in the path from the root to the final node in the destination trie. Thus, a given packet has to be searched for in every source tree of any destination node in the path, from the root to the best destination prefix. 

**Figure 6:**
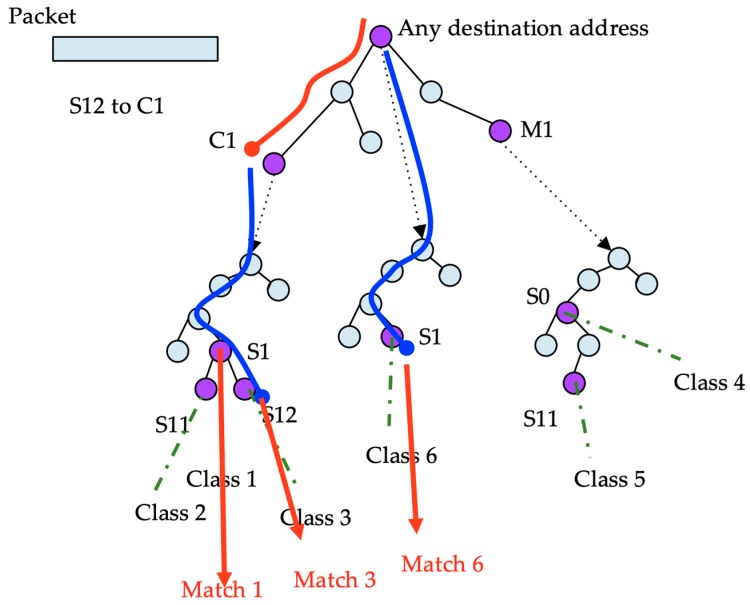
Search procedure example traversing a DStrie.

The classes are stored in only one place in the structure; however, to search for every possible class, multiple source tries must be searched recursively. In the example shown in Figure 6, to classify a packet with source S12 and destination C1, the destination address is searched for in the topmost trie. Two nodes match: the root node corresponding to any address, and the node corresponding to C1. In both nodes, the source trie searched for the source address. In the first one, node S1 points to class six. In the latest, the search in the source trie obtains S1 and S12 pointing to classes three and one, respectively. Thus, the search results in three hits.

The number of operations that is required to match an address in this type of trie depends on the average bit length of the prefixes that are stored in the trie, as this yields the average depth of the binary tree. In our evaluation scenario, a significant amount of full-length prefixes are stored in the destination trie (individual devices), as well as in every source trie. Therefore, the expected time to match an address down a tree should be O(n), where n is the number of bits in network addresses.

The time to process a packet is given by the time that it takes to search a source address in a source tree multiplied by the average number of source tries that have to be traversed, which is also O(n). Therefore, the total time should be in the order of O(n^2).

#### C. Set Pruning DStries

**Figure 7:**
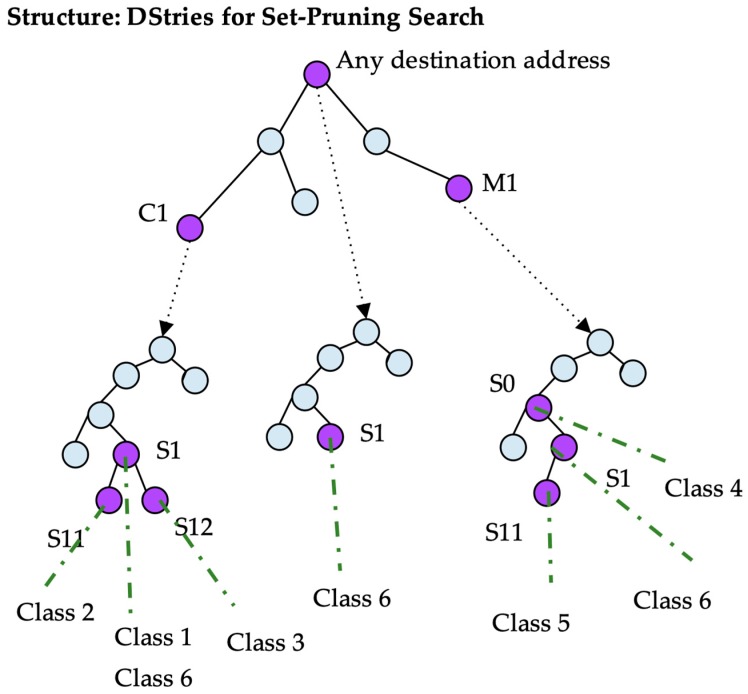
Set-pruning DStries example.

This algorithm presents improvements over DStries with recursive search. A trie is built with every possible destination prefix as before. However, every class is stored not only in the source tree given by its destination, but also in the source tries of its children in the destination trie. A class is stored multiple times, thereby increasing the amount of memory that is used by the data structure. A certain node in a source trie may point to a list of classes instead of a single class. In Figure 7, the source trie pointed to by node C1 contains several classes at node S1.

**Figure 8:**
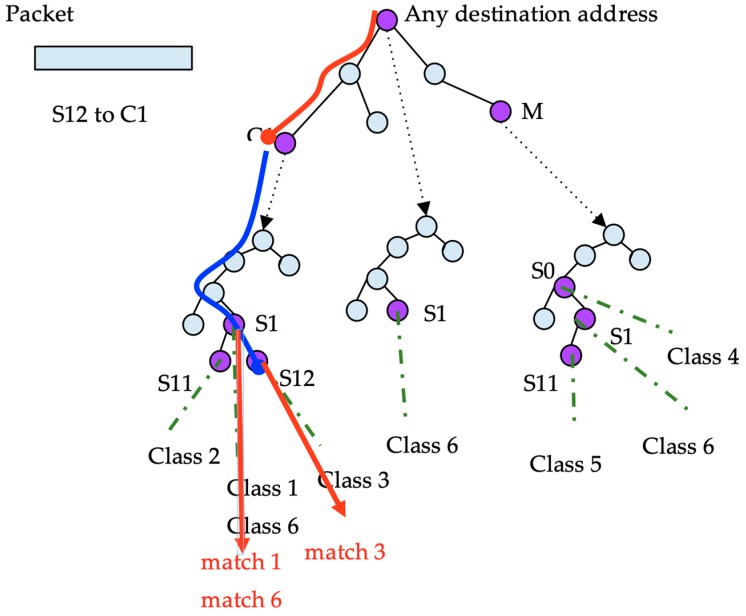
Search example traversing a set-pruning DStrie.

In this algorithm, the search that is undertaken for every packet is simpler than that in DStries with recursive search (see Figure 8). Every packet destination address is searched for the best destination match. Afterwards, the source address is searched for through a single source tree for all of the matches. A single source trie is inspected per packet, but typically, the source tree is denser. The algorithm consumes more memory by storing redundant information to achieve a faster structure traversal.

The expected time to match a packet is in the order of traversing a search tree for the destination address, and subsequently another one for the source address. Therefore, the expected running time is O(2n).

We have implemented all of the algorithms mentioned herein inside a custom time-series computing software that uses a pcap [19] file as input. We use it to evaluate the computing time for several real-world traffic traces.

### 2.3. Network Traffic for Performance Evaluation

To evaluate the performance of these algorithms, network traffic and sets of classes are required. Several traffic captures have been used to provide network packets for classifications (Table 2). The class list to observe is generated from the prefixes and addresses that are present in the captured traffic. The purpose is to generate a realistic scenario. If random prefixes and addresses were generated, most of them would be discarded quickly in the classifier, because they could be found in short branches of the tries. This would yield a false positive bias towards the DStries methods. 

We extract every network address that is present in the traffic trace, as well as every network prefix using prefix length p=24. This list is used to generate every possible combination of a source prefix and destination address, and its corresponding source address and destination prefix. This set of combinations is used as the maximal classes set. To test an algorithm, a subset of $N_{C}$ of these classes is randomly extracted. The trace is processed with this set of classes to evaluate the processing speed. The average number of packets that is processed per second is recorded, as well as the time spent building the classes’ structure into memory and the total memory footprint.

For every classification algorithm, the experiment is repeated using random subsets of classes with different $N_{C}$ sizes.

Table 2 shows the primary statistics from the traffic traces that are used in the evaluation. All of them are IPv4 (n=32 bits) packet traces. Trace upna1h was captured at a university Internet access link supporting the traffic from more than 2000 devices. Trace iot1h was captured at a production IoT network with at least 200,000 operating devices. We captured approximately one hour of traffic at both links. Each of the traces contains more than 50 million packets. When real IoT traffic is not available, the research community resorts to generic IP traffic from desktop computers. Using trace upna1h, we show that the absolute values in the evaluation depend heavily on the traffic pattern. No valid conclusions could have been extrapolated from generic Internet traffic alone. The specific case of traffic from a real IoT scenario must be taken into consideration in the evaluation, as we do.

Both traces contain a large number of different network addresses in use (more than 200,000). Trace iot1h concentrates them into approximately 9000 prefixes (p=24) while upna1h spreads them out over more than 200,000 prefixes. For upna1h, a maximal class set is built, containing 1.19 million classes. For iot1h, the maximal class set contains more than 724,000 classes.

**Figure 9:**
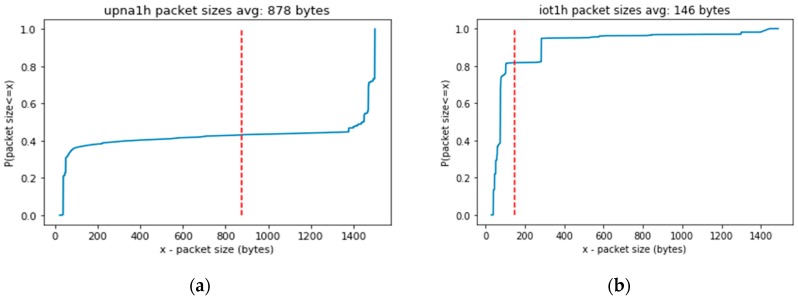
Cumulative distribution functions of packet sizes (**a**) in upna1h traffic trace, or (**b**) in iot1h traffic trace.

Figure 9 shows the different experimental cumulative distribution functions of packet sizes in each of the traffic traces. The average packet size (indicated by the dashed red line) is much larger in a generic Internet access scenario (trace upna1h) than in a real IoT scenario (trace iot1h). These sizes will affect the packet processing speed, as will be shown in the next section.

Traffic processing is performed using a single core in a Xeon E5-2609 CPU at 1.7 GHz with 128 GB of random access memory (RAM). The trace is preloaded in RAM to measure the packet processing time without disk access influence.

## 3. Results and Discussion

**Figure 10:**
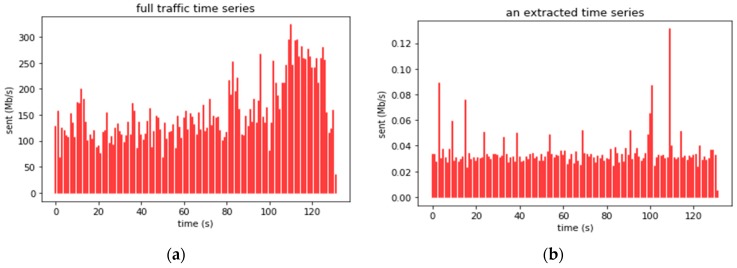
Traffic time series for two minutes (**a**) in the whole trace upna1h, or (**b**) in only one user in trace upna1h.

The input traffic traces were processed by the described algorithms using different random sets of $N_{C}$ classes. Figure 10a shows a two-minute fragment of the time series for the total traffic in trace upna1h. Figure 10b shows the extracted time series for one of the classes. In this section, we evaluate the traffic intensity that a network probe could withstand to create several time series for thousands of IoT devices in real time.

**Figure 11:**
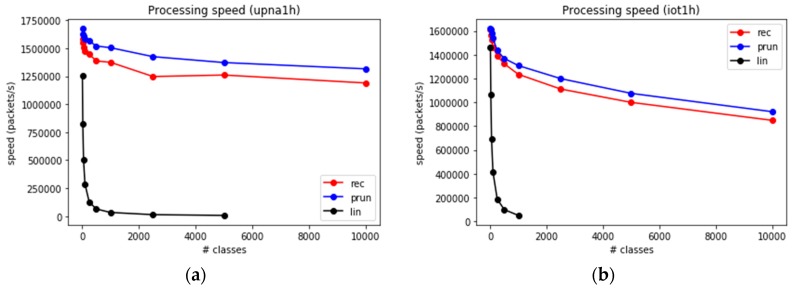
Packet processing speed for small sets of classes. (**a**) upna1h trace (**b**) iot1h trace.

We span a range from hundreds to hundreds of thousands of classes in the set (i.e., number of time series to compute simultaneously). The number of supported devices depends on the number of classes (number of time series) per device. These time series are computed from the input traces using the three presented algorithms. Sets containing up to 5000 classes are evaluated using the three algorithms. Larger sets are simulated using only the fastest methods. The throughput obtained in packets per second, using up to 5000 classes, is shown in Figure 11a (for upna1 trace) and Figure 11b (for iot1h trace).

The throughput depends strongly on $N_{C}$. For the linear search algorithm, the computation time increases as $O\left(N_{C}\right)$; therefore, the throughput decreases proportional to 1NC. This is an extremely fast decay compared to the other algorithms, thereby causing the LS to be non-competitive for thousands or more classes. 

Recursive search and set-pruning DStries algorithms decay more slowly, and achieve more than one million packets/s for thousands of classes.

**Figure 12:**
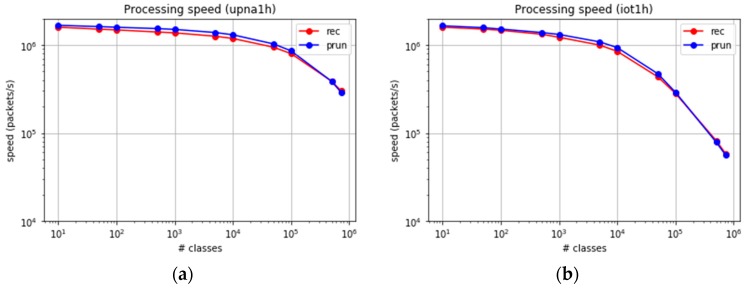
Packet processing speed for large sets of classes. (**a**) upna1h trace. (**b**) iot1h trace.

The experiment was extended to at least 724,000 classes, excluding the computation of the LS algorithm, whose results are predicted easily. Figure 12a,b show the packet processing speed for both traces.

**Figure 13:**
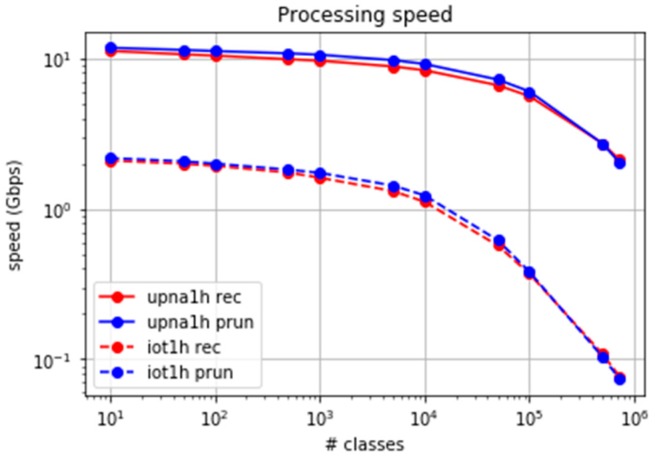
Processing speed in bits per second comparison for upna1h and iot1h traces.

Both algorithms present a similar behaviour in the number of processed packets per second, with a slightly higher performance in the case of set-pruning DStries. The throughput in gigabytes (Gb)/s depends on the average packet size. Figure 13 shows the achievable processing bit rate when considering the average packet sizes. The average per packet processing time in our reference probe is about four microseconds when 100,000 classes are used, and less than 18 microseconds for 700,000 classes. Less than 1% of the packets suffer a processing time larger than 200 microseconds, even when 700,000 classes are used. Therefore, the error margin in time-series computation with samples every second is low.

The upna1h traffic trace presents average Internet packet sizes; therefore, the multigigabit per-second processing speeds are reached. Trace iot1h was obtained from a real IoT scenario, where the packets were smaller than the average sizes in the Internet. For a similar number of processed packets per second, a much smaller traffic bitrate was consumed (approximately one Gb/s). We found it important to consider the specific traffic characteristics in real IoT traffic.

From these results, we can estimate the number of IoT devices that a single-core CPU could analyse. The limit depends on the traffic behaviour; therefore, we have included results based on a typical IoT profile (extracted from trace iot1h), and on a generic Internet access profile (extracted from trace upna1h). 

A single point in Figure 12 represents the maximum average packet arrival rate that could be processed for a specific number of classes, $N_{C}$. This number of classes is simply the number of IoT devices multiplied by the number of time series per device. The maximum packet arrival rate corresponds to the aggregate of traffic from the same number of devices. Assuming certain traffic intensity or an average per-device packet arrival rate, the maximum number of devices can be obtained. We introduce this intensity as a parameter using the average number of packets per second sent by each device. For example, a sensor that collects temperature measures and contacts its central repository once per minute, sending all of the measures with the exchange of 12 network packets generating 12/60 = 0.2 packets per second (pkt/s).

**Figure 14:**
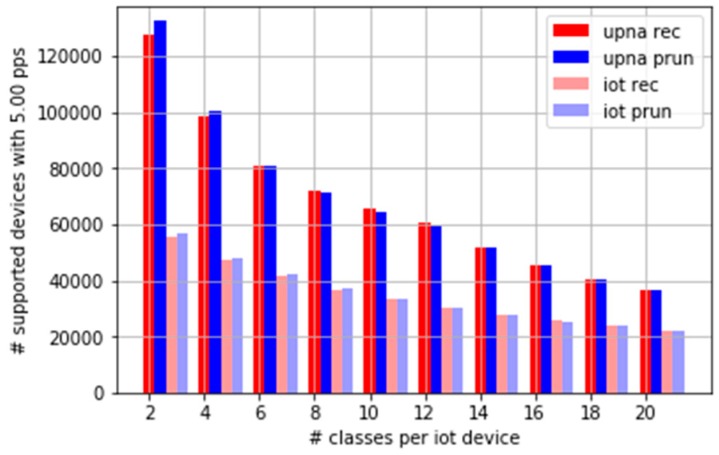
Number of supported devices that generate an average of five pkt/s.

Figure 14 shows the maximum number of devices supported in real time versus the number of time series to compute per device. As a bidirectional time series requires two classes, the case of six time series (six classes) corresponds to three bidirectional time series. This could represent, for example, the case of an operator who wants to measure the bidirectional traffic from each IoT device to its central collector, any server in its server farm, and everywhere else. The results for 10 time series consider the possibility of two extra bidirectional time series per device. We have included the results for up to 20 classes per device, or 10 bidirectional time series. Figure 14 is based on the devices that generate an average of five pkt/s.

The results depend on the traffic profile, or how the random traffic process is generated. For a present IoT network scenario with six classes per device, and an average of five pkt/s per device, more than 40,000 devices are supported. Using 20 classes, at least 20,000 devices are supported in a single-core CPU processing machine. For a traffic profile similar to a generic Internet access link, better results are obtained, especially for low numbers of classes. The results for typical IoT scenarios are worse, owing to the small average packet sizes.

**Figure 15:**
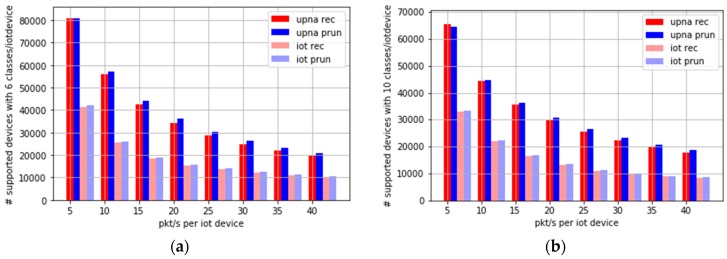
Number of devices supported, depending on the traffic generated per IoT device (**a**) extracting 6 time series per device, or (**b**) extracting 10 time series per device.

Figure 15a,b show the number of supported devices when the amount of traffic generated by each device is increased from five pkt/s up to 40 pkt/s. Figure 15a considers six time series per device, and Figure 15b shows the requirement of 10 time series per device. This represents future scenarios where IoT devices collect more frequent measurements or send larger files to the central collector. For example, a camera creating a 15-kB image every second could easily result in a traffic intensity of 15 or 20 pkt/s, considering both directions of the traffic.

Figure 15 shows that by using six classes (three bidirectional time series) and high traffic intensity-generating devices (up to 40 pkt/s), at least 10,000 devices would be supported by both algorithms.

**Figure 16:**
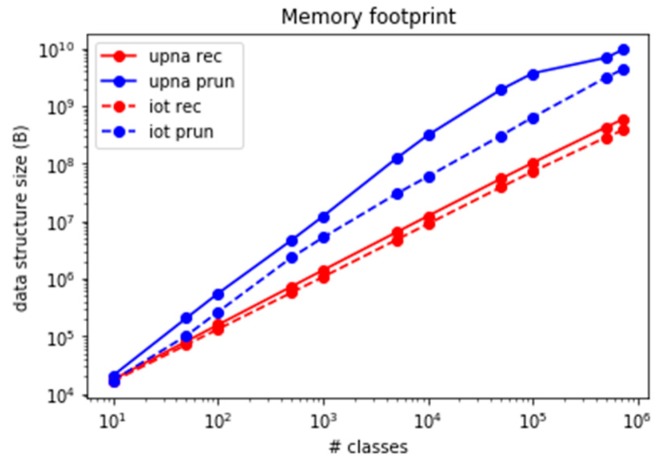
Comparison of memory usage.

Although both set-pruning methods (DStries and DStries with recursive search) offer similar speed results, they present some hidden drawbacks that must be considered. The data structure that set-pruning DStries stores in memory duplicates the class information; therefore, its memory footprint is higher than the one from a simple recursive search or the linear search method. Figure 16 shows the memory usage for both DStries-based algorithms and both traffic traces. Although the linear method presents minimal memory usage, it must be discarded as a suitable algorithm, owing to its low performance in terms of packets processed per second for a large number of classes. 

**Figure 17:**
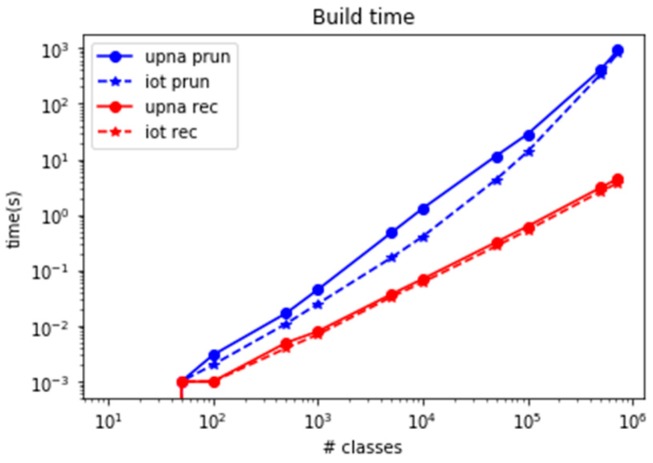
Comparison of required time to add a new class.

The second drawback in set-pruning DStries is the difficulty in updating its memory structures. Whenever a new class has to be added to the structure, the whole structure must be recreated from scratch. This is simpler than updating the structure incrementally. Adding (or removing) classes must be performed whenever new IoT devices are added to the network and monitoring platform. This is typical in large deployment scenarios, as it could be in household metering devices in an electric company. Figure 17 shows the time that is required to build the DStrie in the reference computer when a single new class must be added to an already existing structure. The time to build a set-pruning DStrie for hundreds of thousands of classes may reach several minutes; thus, the algorithm is not useful if the set of classes to monitor must change frequently.

## 4. Conclusions

We herein demonstrated how a single-core CPU could create the traffic time series for tens of thousands of IoT devices, even when several time series were required for each device. The results depended on the number of time series desired, and the traffic intensity created by each IoT device. Considering different traffic profiles, the results were validated using traffic from a real IoT deployment scenario with more than 200,000 devices, and from a generic Internet access link.

The algorithms of linear search, DStries with recursive search, and set-pruning DStries, were evaluated. Although the linear search algorithm presented the simplest implementation and the lowest memory requirements, it provided the worst results regarding the number of time series that it could compute in real time compared to the other algorithms. It could only be used in extremely small scenarios. Both DStries with recursive search and set-pruning DStries yielded similar results in computation speed, and hence the number of devices supported in real time. DStries with recursive search exhibited lower memory requirements compared to set-pruning DStries; therefore, it is better suited for scenarios with low computing power and memory available in each time-series computing node. Set-pruning DStries also created complex in-memory structures, and required more time when a new IoT device had to be added or removed. For highly dynamic scenarios where new nodes are added frequently, DStries with recursive search is the most suitable algorithm, as it offers the lowest memory footprint and the lowest modification time. In a single-core CPU, it can create three bidirectional time series for each one of more than 20,000 IoT devices in real time, when each device sends an average of 10 packets per second.

## Figures and Tables

**Table 1:** Class definitions for the examples in the algorithm and description of the data structures.

#class	Source	Destination	
1	S1	C1	Traffic from sensor group S1 to controller C1
2	S11	C1	Traffic from sensor S11 to controller C1
3	S12	C1	Traffic from sensor S12 to controller C1
4	S0	M1	Traffic from any sensor to malware host M1
5	S11	M1	Traffic from sensor S11 to malware host M1
6	S1	Any	Traffic from sensor group S1 to anywhere

**Table 2:** Global statistics from the evaluation traffic traces.

trace	duration	#packets	bytes	throughput	average packet size	#unique address	#prefixes 24 bits	#classes
upna1h	1 h	193 M	170 GB	381 Mbps	~880 B	290 k	211 k	1193 k
iot1h	1 h	58 M	9 GB	24 Mbps	~165 B	214 k	8.9 k	724 k
